# Exploring the role of GhN/AINV23: implications for plant growth, development, and drought tolerance

**DOI:** 10.1186/s13062-024-00465-2

**Published:** 2024-03-14

**Authors:** Kaikai Qiao, Qingtao Zeng, Jiaoyan Lv, Lingling Chen, Juxin Hao, Ding Wang, Qifeng Ma, Shuli Fan

**Affiliations:** 1 National Nanfan Research Institute (Sanya), Chinese Academy of Agricultural Sciences, 572024 Sanya, Hainan China; 2 National Key Laboratory of Cotton Bio-breeding and Integrated Utilization, Institute of Cotton Research of Chinese Academy of Agricultural Sciences (CAAS), 455000 Anyang, Henan China; 3 The 7th Division of Agricultural Sciences Institute, Xinjiang Production and Construction Corps, 833200 Kuitun, Xinjiang China; 4 Anyang Academy of Agricultural Sciences, 455000 Anyang, Henan China; 5 Anyang Meteorological Service, 455000 Anyang, Henan China

**Keywords:** *GhN/AINV23*, Bioinformatics, Subcellular localization, Plant growth, Drought stress

## Abstract

**Background:**

Neutral/alkaline invertases (N/AINVs) play a crucial role in plant growth, development, and stress response, by irreversibly hydrolyzing sucrose into glucose and fructose. However, research on cotton in this area is limited. This study aims to investigate *GhN/AINV23*, a neutral/alkaline invertase in cotton, including its characteristics and biological functions.

**Results:**

In our study, we analyzed the sequence information, three-dimensional (3D) model, phylogenetic tree, and cis-elements of *GhN/AINV23*. The localization of GhN/AINV23 was determined to be in the cytoplasm and cell membrane. Quantitative real-time polymerase chain reaction (qRT-PCR) results showed that *GhN/AINV23* expression was induced by abscisic acid (ABA), exogenous sucrose and low exogenous glucose, and inhibited by high exogenous glucose. In *Arabidopsis*, overexpression of *GhN/AINV23* promoted vegetative phase change, root development, and drought tolerance. Additionally, the virus-induced gene silencing (VIGS) assay indicated that the inhibition of *GhN/AINV23* expression made cotton more susceptible to drought stress, suggesting that GhN/AINV23 positively regulates plant drought tolerance.

**Conclusion:**

Our research indicates that *GhN/AINV23* plays a significant role in plant vegetative phase change, root development, and drought response. These findings provide a valuable foundation for utilizing *GhN/AINV23* to improve cotton yield.

**Supplementary Information:**

The online version contains supplementary material available at 10.1186/s13062-024-00465-2.

## Introduction

Sugars exert an influence on every aspect of the growth process in plants [1, 2]. Sucrose is synthesized in the leaves and then transported to various organs or tissues through either symplastic or apoplastic translocation [3]. Besides acting as a source of energy, sucrose also functions as a signaling molecule, exerting control not only over plant metabolism, but also over plant development [4, 5]. Among them, sucrose and glucose have a significant impact on the development of the root system. Freixes et al. (2002) proved that sucrose can promote taproot elongation in a dose-dependent manner, with a higher concentration of exogenous sucrose resulting in faster taproot elongation [6]. *Arabidopsis* seedlings treated with exogenous sucrose exhibited an increase in lateral root number, which was found to be directly related to the sugar content in the roots [6]. Similarly, exogenous glucose can also promote taproot growth [7], but high concentrations can be detrimental to taproot growth by inhibiting meristem [8], exerting a dose effect. Roots are crucial for plant growth, and plant relies on roots to obtain nutrients to adapt to environmental stress in soil [9]. These studies indicate that sucrose or glucose can respond to stress by affecting root development.

Sucrose is broken down into glucose and fructose by sucrose synthase and invertase, with invertase being further categorized into acid invertase (AINV) and neutral/alkaline invertase (N/AINV) based on optimum pH [10]. N/AINVs have been confirmed to participate in various stages of the plant life cycle, particularly in stress response and root development [11–18]. The gene *MeNINV1*, an alkaline/neutral invertase gene derived from *Manihot esculenta*, could enhance sucrose catabolism and promote vegetative growth in transgenic *Arabidopsis* plants [19]. Inhibition of the expression of *NtNINV10* decreased the glucose and fructose in tobacco leaves [20]. *INVAN6* plays a key role in the regulation of male meiosis in maize [21]. The high expression of *TaCWI50* and *TaVI27* may increase wheat thousand grain weight [22]. Drought resistance in rice was improved by increasing the root-to-shoot ratio and inducing root N/AINV activity [23]. Two AT-Hook proteins mediated sucrose metabolism by regulating the expression of *A/NINV7* to enhance the cold tolerance of *Poncirus trifoliata* [24]. Similarly, osmotic stress led to *AtN/AINVs* expression and soluble sugar accumulation in *Arabidopsis* in order to maintain the respiratory electron transport chain and reactive oxygen species homeostasis [13, 25, 26]. Further, *PtrN/AINV* overexpression in *Poncirus trifoliata* lowered reactive oxygen species content, increased photosynthetic capacity, reduced oxidative damage, and decreased water loss rate, all of which led to enhanced plant stress resistance [27]. *TaN/AINV1* negatively regulated stripe-rust resistance in wheat by enhancing carbohydrate accumulation and reducing photosynthesis in damaged tissues [28]. In terms of root development, the *Oscyt-inv1* rice mutant showed short roots, delayed flowering, and partial sterility [29]. A single mutant of *cinv1* in *Arabidopsi*s exhibited a shortened taproot and insensitivity to lateral root growth induced by osmotic stress [13]. Lack of *cinv1/cinv2* genes resulted in decreased N/AINV activity, enlarged cells in the root elongation region, and shortened taproot [14, 30]. Additionally, phosphatidylinositol monophosphate 5-kinase 9 (PIP5K9) can inhibit taproot development by reducing AtN/AINV activity via direct N/AINV interaction [18].

Cotton is an essential raw material in the textile industry, contributing significantly to the global economy [31]. However, its output and quality continue to face limitations due to adverse environmental conditions [32]. In previous studies, we reported on the *N/AINV* gene family in cotton and analyzed the function of *GhN/AINV13*, revealing its regulation of drought resistance through interaction with Gh14-3-3 [33]. Additionally, while the capability of *GhN/AINV23* to hydrolyze sucrose was preliminarily verified *via* yeast complementation [33], its biological function remained unexplored. In this study, we report on the characteristics and subcellular localization of *GhN/AINV23*. We demonstrated that overexpressing *GhN/AINV23* promotes *Arabidopsis* root development, which is regulated by sucrose and glucose. Additionally, GhN/AINV23 positively regulates drought resistance in plants. These findings provide a foundation for future applications of this gene in cotton production.

## Materials and methods

### Bioinformatics analysis

Gene information was obtained from CottonFGD [34]. The DNAMAN software was utilized to perform molecular weight (MW), isoelectric point (pI), and amino acid sequence consistency analysis. The 3D structures were analyzed by PHYRE server v2.0 (http://www.sbg.bio.ic.ac.uk/phyre2/html/page.cgi?id=index). Phylogenetic tree was established using MEGA 7.0 software with neighbor-joining method [35]. The cis-element was analyzed using the PlantCARE online tool [36] and then created the image using TBtools software [37].

### Subcellular localization

We cloned the coding sequence (CDS) of *GhN/AINV23* from TM-1 cDNA and linked it with the pCAMBIA-2300-35 S::eGFP vector, producing the pCAMBIA-2300-35 S::GhN/AINV23-eGFP vector. The two vectors were transferred into the *Agrobacterium tumefaciens* strain GV3101, then injected into tobacco leaves with needle. The inoculated tobacco plants were cultured for 12 h dark/24 h light. Moreover, further subcellular localization was determined using *Arabidopsis* protoplasts. The plasmid was extracted using the EndoFree Maxi Plasmid Kit (TIANGEN, Beijing) to ensure high quality and concentration. The extraction and transformation of *Arabidopsis* young leaves protoplasts were performed using the *Arabidopsis* protoplasm preparation and transformation kit (Coolaber, Beijing). After light-culturing for 24 h, we observed the transformed protoplasts under a Dmi8 inverted microscope (Leica, Wetzlar, Germany) to visualize the eGFP fluorescence.

### Cotton materials and treatment

The cotton material used in this experiment was CCRI24, which was grown in a greenhouse at 28 °C, 16 h of light, 8 h of darkness and 80% relative humidity. We treated 20-day-old plants with 100 ABA (abscisic acid) and measured the expression level of *GhN/AINV23* in the second true leaf at 0, 3, 6, 9, 12, 24, and 48 h. Additionally, we moved sterilized cotton seeds to MS medium containing 0, 10, 20, 30, or 60 g/L sucrose or glucose, and then we collected the roots for gene expression analysis after 14 days.

### Transgenic *Arabidopsis* and treatment

To construct 35 S: GhN/AINV23, we cloned the CDS of *GhN/AINV23* into the pBI121 vector containing the 35 S promoter. The 35 S: GhN/AINV23 vector was transferred into *Arabidopsis* using the *Agrobacterium*-mediated floral dip method. After approximately 7 weeks, *Arabidopsis* seeds were harvested. For screening positive transgenic lines, we used 1/2 MS medium containing 50 mg/L kanamycin, followed by continual validation through qRT-PCR. T3 homozygous lines of three transgenic lines of overexpressing *GhN/AINV23* were selected for further analysis. The Eloam high-speed photographic apparatus (Eloam, Shenzhen) and WSeen SC-G software (WSeen, Hangzhou) were used for photographing, calculating leaf area, and measuring the length-to-width ratio of *Arabidopsis* leaves. We measured neutral invertase activity, sucrose, glucose, and fructose using the following assay kits: Neutral invertase Assay kit (Solarbio® BC0570) [38], Plant Tissue Sucrose Content Assay Kit (Solarbio® BC2460) [39], Glucose Assay kit (Solarbio® BC2500) [40], and Tissue Fructose Assay Kit (Solarbio® BC2450) [41]. The measurements were taken by visible spectrophotometry. Solutions containing sucrose or glucose at mass ratios of 1%, 2%, and 3% were added to 1/2 MS medium for sugar treatment purposes. Similarly, mannitol with 1%, 2% and 3% mass ratio were added to simulate drought treatment. Meanwhile, plants with similar growth in soil were selected for drought stress, and the performance was observed after 15 days, during which the flower stamens were cut. Plant survival rates were observed after rehydration for 7 days.

### RNA extraction and qRT-PCR

RNA was successfully extracted from cotton leaves and roots using the FastPure® Plant Total RNA Isolation Kit (Vazyme, Nanjing). RNA extraction from Arabidopsis leaves using RNAprep Pure Plant Plus Kit (TIANGEN, Beijing). The cDNA was obtained by TransCript All-in-One First-Strand cDNA Synthesis SuperMix for qPCR (One-Step gDNA Removal) Kit (TransGen Biotech, Beijing). A quantitative real-time polymerase chain reaction (qRT-PCR) was performed by ABI 7500 realtime PCR System and MonAmp SYBR Green qPCR Mix (Monad, China). All primers used are listed in Table S1. Experimental data were analyzed using the 2^-△△CT^ method [42]. All experiments were carried out using three independent biological replicates and three technical replicates. The measured data were statistically analyzed using a one-way analysis of variance (ANOVA) and the Student’s t-test to determine statistically significant differences between groups. The significance level was set at *P ≤ 0.05 and **P ≤ 0.01 to indicate significant differences.

### Virus-Induced Gene silencing (VIGS)

The tobacco rattle virus (TRV) vectors were used to perform virus-induced gene silencing (VIGS) assays [43]. The TRV system is comprised of two vectors: pTRV1 (pYL192) and pTRV2 (pYL156). We inserted a 236 bp specific fragment of the GhN/AINV23 gene into the pTRV2 vector to construct the pTRV2: GhN/AINV23. The pTRV2: GhN/AINV23, pTRV2:00, pTRV2:CLA1 (positive control), and pTRV1 were transformed into *Agrobacterium tumefaciens* strain LBA4404 and placed in 24 °C constant temperature shake for 12–24 h. After centrifugation (28 °C, 4000 rpm), the strain was suspended in an osmotic medium containing 10 mM MgCl_2_, 10 mM MES, 5% sucrose solution, and 20 µM acetosyringone (AS) to an OD 600 of 1.5, followed by placing it in the dark for 2–3 h. The *Agrobacterium tumefaciens* culture medium containing pTRV2: GhN/AINV23, pTRV2:00, and pTRV2:CLA1 were mixed with pTRV1 at a 1:1 ratio, then we injected it into CCRI24 cotyledons and placed them in the dark for 12–24 h. All plants were then grown under normal controlled conditions (16 h of light/8 hours of dark, 24 °C). The plants were grown for 20 days and then subjected to water shortage treatment for 10 days. Catalase (CAT) and malondialdehyde (MDA) levels were evaluated using the Catalase (CAT) and Malondialdehyde (MDA) Assay Kit (Solarbio, Beijing, China).

## Results

### Bioinformatics analysis of *GhN/AINV23*

To investigate the function of the *GhN/AINV23* gene, we conducted a bioinformatics analysis based on previous research [33]. The ID of gene is GH_D09G1278, the gDNA is 5272 bp, and the full-length CDS is 1680 bp. The molecular weight (MW) and isoelectric point (pI) of *GhN/AINV23* are 63919.6 da and 6.36, respectively. The three-dimensional (3D) model exhibited that GhN/AINV23 contains 12 α helixs and 2 β sheets (Figure S1A).

The phylogenetic tree analysis indicated GhN/AINV23, AtCINV1 and AtCINV2 were gathered together (Figure S1B). The amino acid sequence consistency of GhN/AINV23 and AtCINV1, AtCINV2 was 81.43%, 85.23%, respectively (Figure S1C). To better understand the regulatory relationship of *GhN/AINV23*, we obtained a 2000-bp upstream sequence of the gene and performed cis-element analysis (Figure S1D). The result indicated that *GhN/AINV23* promoter region contains many hormones and stress-related elements such as drought response element (MBS), temperature response element (LTR), anaerobic induction element (ARE), gibberellin-responsive element (P-box), auxin-responsive element (TGA-element), MeJA-responsive element (TGACG motif), salicylic acid (SA) response element (TCA-element), ethylene-responsive element (ERE) and ABA-responsive element (ABRE).

### GhN/AINV23 was localized in the cytomembrane and cytoplasm

**Fig. 1:**
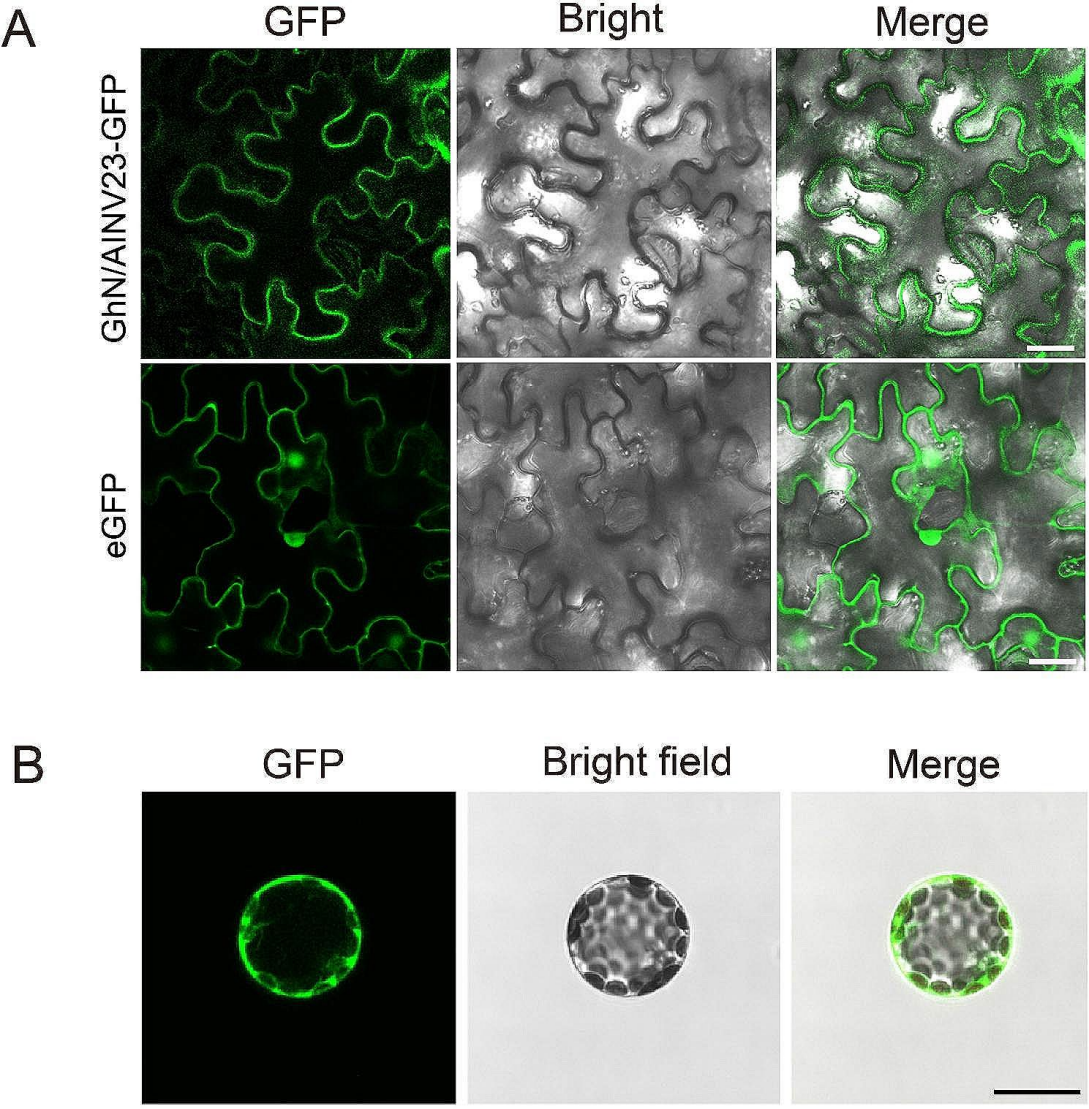
Subcellular localization of GhN/AINV23 in tobacco (*Nicotiana benthamiana*) leaves (**A**: Scale bar = 50 μm) and *Arabidopsis* protoplasts (**B**: Scale bar = 25 μm)

The subcellular location of a protein is an important indicator of its function [44]. In our study, we constructed GhN/AINV23-GFP fusion proteins, which were expressed in tobacco leaves. The fluorescence microscopy images showed that the green fluorescence was concentrated at the cell edge (Fig. 1A), indicating that GhN/AINV23 was localized to the cell membrane. To further confirm the subcellular location of GhN/AINV23, we transformed the constructed vectors into the protoplasts of *Arabidopsis thaliana*. The results showed that the green fluorescence was mainly concentrated in the cell membrane and cytoplasm (Fig. 1B), indicating that *GhN/AINV23* function in the cell membrane and cytoplasm.

### Expression patterns of *GhN/AINV23* under ABA, sucrose, and glucose treatment

**Fig. 2:**
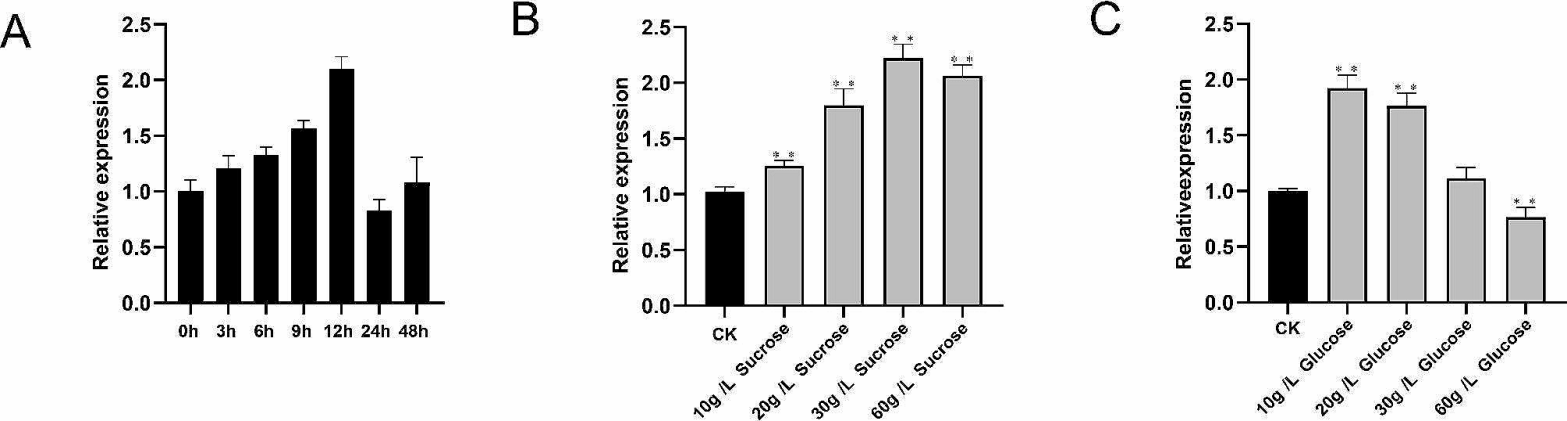
Expression level of *GhN/AINV23* after ABA treatment (**A**), sucrose treatment (**B**), and glucose treatment (**C**) by qRT-PCR.

To investigate whether *GhN/AINV23* participates in stress response, we observed its expression after ABA treatment. The qRT-PCR results revealed that *GhN/AINV23* expression could be induced by ABA, with its expression showing continuous increases from 0 to 12 h of treatment (Fig. 2A). Given that the neutral-alkal invertase plays a crucial role in sugar metabolism, we tested whether *GhN/AINV23* was involved in sugar metabolism by detecting its expression after sucrose and glucose treatment. Our results showed that *GhN/AINV23* expression increased with the external application of sucrose, reaching a maximum at 30 g/L (Fig. 2B). Similarly, after external application of glucose, the gene expression of *GhN/AINV23* peaked at 10 g/L, and increased glucose concentrations inhibited the expression of *GhN/AINV23* (Fig. 2C).

### Overexpressing of *GhN/AINV23* promoted the growth of *Arabidopsis thaliana*

**Fig. 3:**
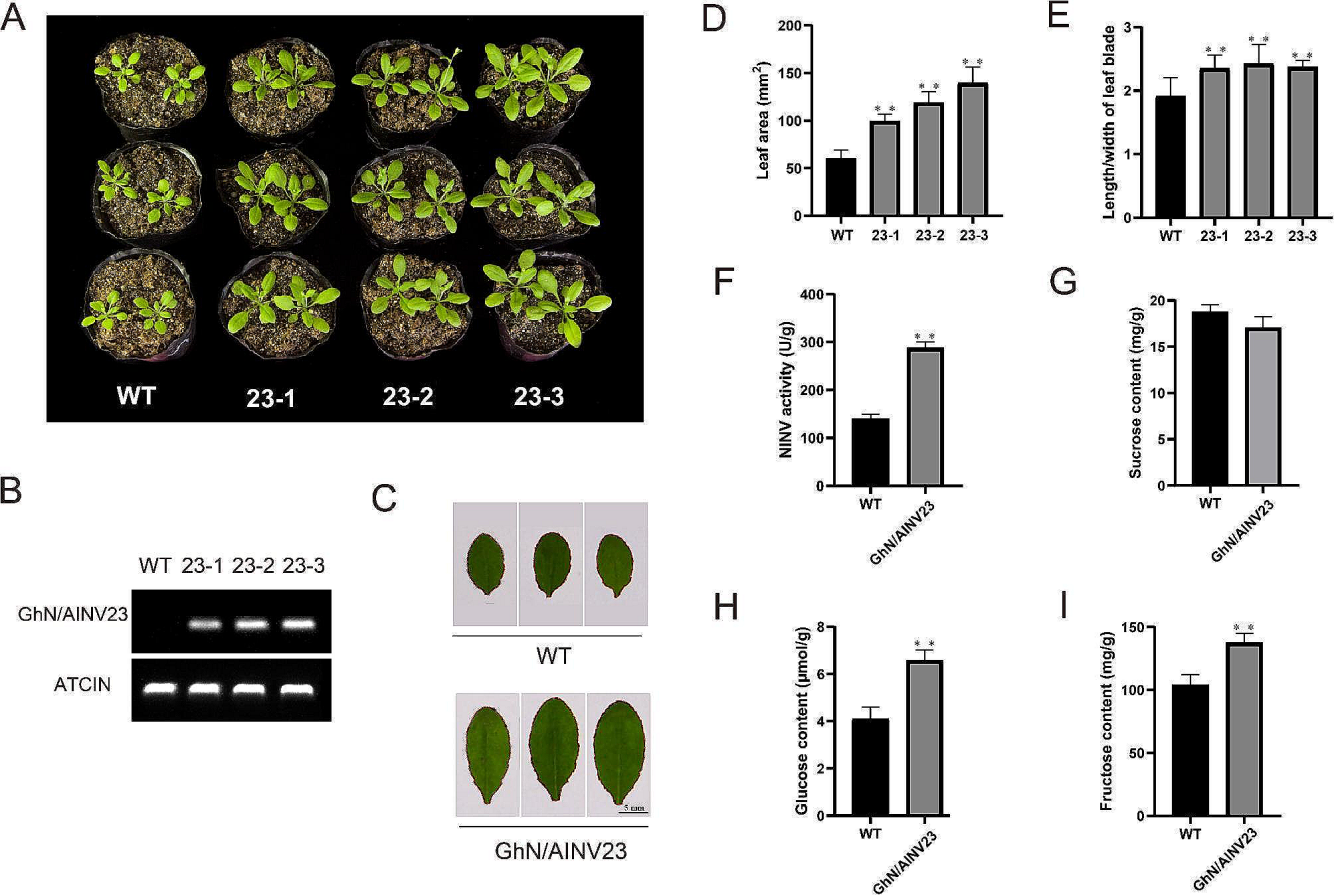
Overexpression of *GhN/AINV23* in *Arabidopsis.* The state of WT plants and transgenic lines (23 − 1, 23 − 2 and 23 − 3) after 21 days of growth (**A**). Semi-quantitative RT-PCR detection of *GhN/AINV23* expression in three independent *Arabidopsis* transgenic lines (**B**). The leaf phenotypes of WT plants and transgenic lines after 21 days of growth (**C**). The leaf area of WT plants and transgenic lines (**D**). The length/width of leaf blade area of WT plants and transgenic lines (**E**). The NINV activity of WT plants and transgenic lines **(F)**. The sucrose content of WT plants and transgenic lines (**G**). The glucose content of WT plants and transgenic lines (**H**). The fructose content of WT plants and transgenic lines (**I**)

To examine the role of *GhN/AINV23* on plant growth and development, we selected three transgenic single-copy homozygous lines (23 − 1, 23 − 2, and 23 − 3) for further analysis (Fig. 3A). The Semi-quantitative RT-PCR results showed that the expression of *GhN/AINV23* in the transgenic lines was higher than that in WT plants (Fig. 3B). We planted transgenic *Arabidopsis* seeds and WT seeds in a culture chamber and observed the phenotype after 21 days. The results showed that the leaves of the transgenic lines were significantly larger than those of the WT, with increases in leaf area (Fig. 3D) and length/width of leaf blade (Fig. 3E).

Furthermore, we detected the N/AINVs activity, sucrose content, glucose content, and fructose content of transgenic *Arabidopsis* and WT plants (Fig. 3F-I). The results showed that N/AINVs activity, glucose content, and fructose content of transgenic lines were higher than those of WT, while sucrose content did not show any significant difference. These results indicate that *GhN/AINV23* affects *Arabidopsis* growth and development by participating in sucrose metabolism.

### Promotion of root development, sensitivity to exogenous glucose, and enhanced drought resistance in *Arabidopsis* by overexpression of *GhN/AINV23*

**Fig. 4:**
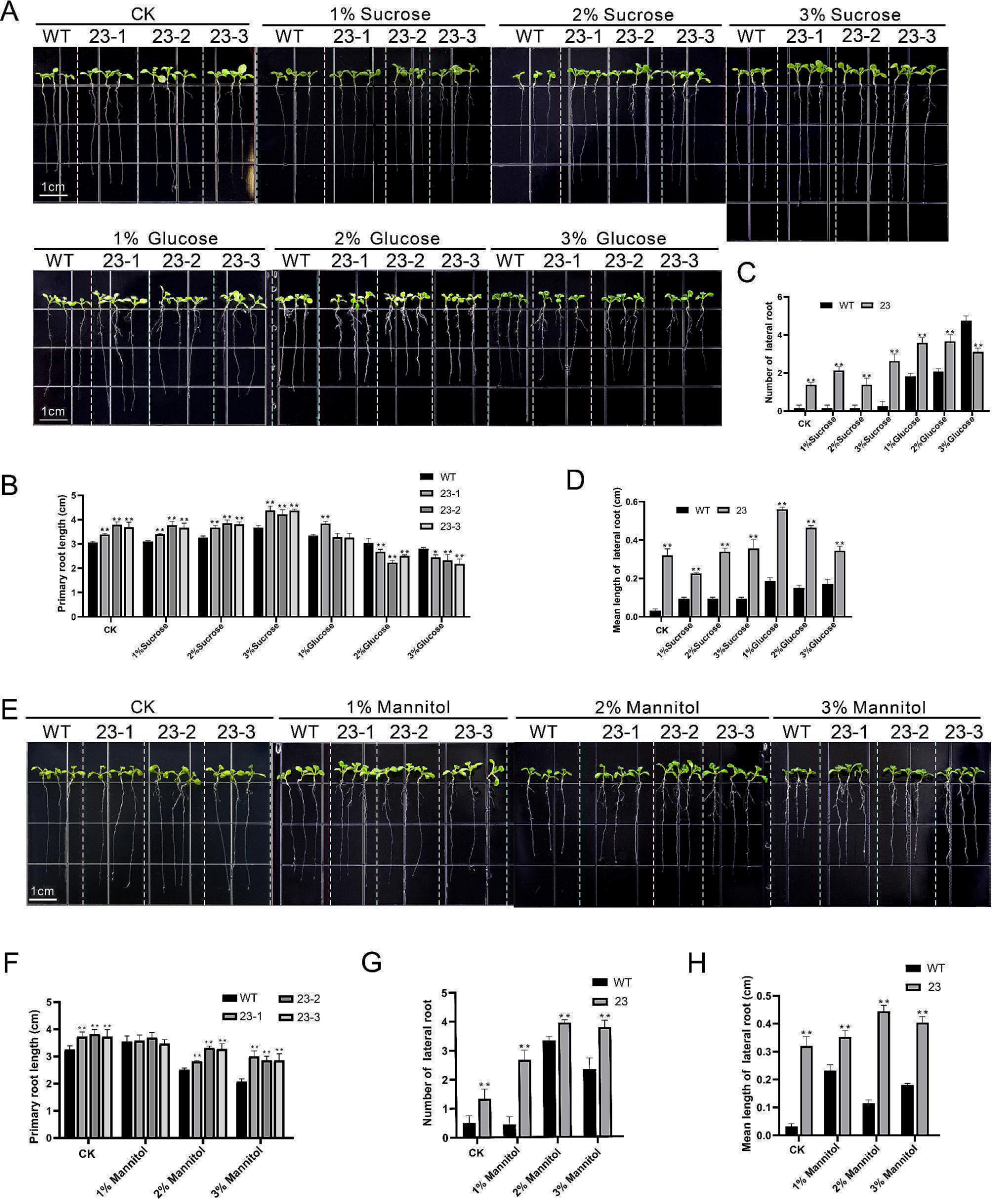
Observation of roots in WT plants and transgenic lines (23 − 1, 23 − 2 and 23 − 3) under different conditions. The root phenotypes (**A**), primary root length (**B**), number of lateral root (**C**) and mean length of lateral root (**D**) of WT plants and transgenic lines under normal 1/2MS medium or 1/2MS medium containing different concentrations of sucrose and glucose. The root phenotypes (**E**), primary root length (**F**), number of lateral root (**G**) and mean length of lateral root (**H**) of WT plants and transgenic lines under normal 1/2MS medium or 1/2MS medium containing different concentrations of mannitol

The previous analysis revealed that GhN/AINV23 is involved in sugar metabolism. To further understand its role in sucrose metabolism, we observed the root growth of transgenic *Arabidopsis* lines and WT after treatment with different sucrose and glucose content. Our results showed that the transgenic lines had longer primary root length and significantly higher lateral root number than the WT in normal 1/2MS medium (Fig. 4A). With increasing sucrose content, both primary root length and lateral root number increased in all lines (Fig. 4B-C), with the overexpressing *GhN/AINV23* transgenic *Arabidopsis* showing significantly greater increases in primary root length than WT (Fig. 4D). However, increasing glucose content inhibited the elongation of primary roots and reduced the number of lateral roots in overexpressing plants compared with WT plants (Fig. 4B-C). These findings indicate that overexpression of *GhN/AINV23* promotes root development in *Arabidopsis* and enhances root sensitivity to exogenous glucose.

The study above indicates that the promoter of *GhN/AINV23* contains drought-responsive elements, and the expression of *GhN/AINV23* is induced by ABA. To investigate whether *GhN/AINV23* is involved in drought stress, we simulated different levels of drought stress with different content of mannitol (Fig. 4E). Our results indicated that primary root growth in all plants was inhibited on the media with mannitol (Fig. 4F), whereas the number and mean length of lateral roots were increased (Fig. 4G-H). Nonetheless, the primary growth of overexpressed plants suffered less inhibition than that of wild-type plants. To further investigate the tolerance of *GhN/AINV23* to drought in *Arabidopsis thaliana* at maturity, we selected 21 days old plants that were treated with water shortage (Fig. 2SA). 15 days later, we found that all WT plants were withered, while transgenic plants grew inhibited (Figure S2B). After being re-watered for 7 days, transgenic plants restored normal growth, but all WT plants had died completely (Figure S2C). These results indicate that overexpressing *GhN/AINV23* enhances drought resistance of *Arabidopsis thaliana*.

### The drought resistance of cotton was reduced after silencing *GhN/AINV23*

**Fig. 5:**
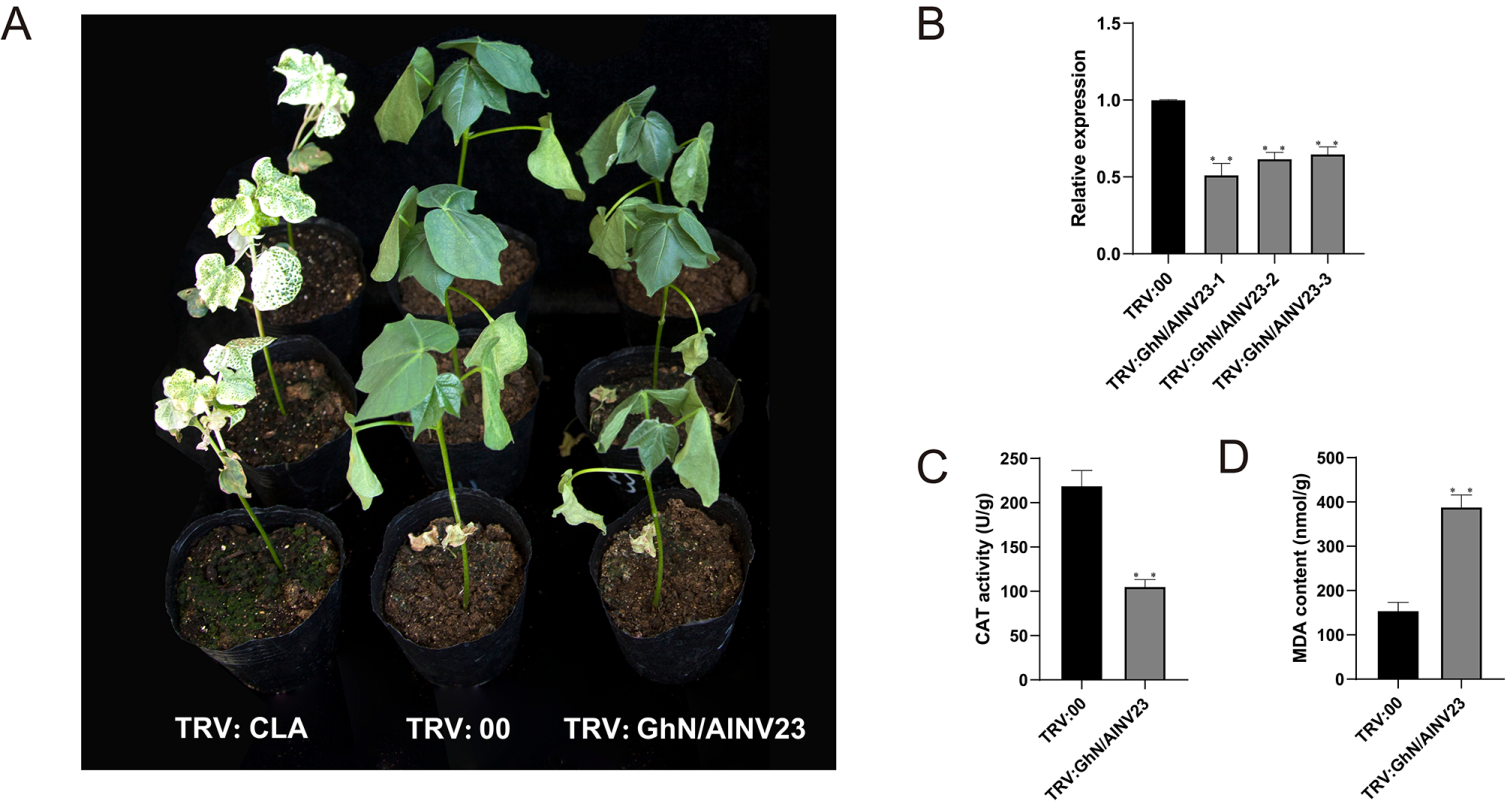
Silencing of *GhN/AINV23* gene reduces cotton drought resistance. Phenotypes of positive control (TRV: CLA) plants, the blank control (TRV:00) plants, and *GhN/AINV23*-silenced plants (**A**). The expression level of *GhN/AINV23* in the blank control and *GhN/AINV23*-silenced (TRV: GhN/AINV23) plants (**B**). The catalase (CAT) activity in the blank control and *GhN/AINV23*-silenced plants (**C**). The malondialdehyde (MDA) content in the blank control and *GhN/AINV23*-silenced plants (**D**)

We used the virus-induced gene silencing (VIGS) technique to investigate the role of *GhN/AINV23* in the stress resistance process of cotton. The positive plants exhibited an albino phenotype (Fig. 5A), and *GhN/AINV23* expression was successfully inhibited in TRV: GhN/AINV23 plants (Fig. 5B), indicating the success of the experimental system. After 10 days of water shortages, we observed significant wilting in the *GhN/AINV23*-silenced plants compared to TRV:00 plants (Fig. 5A). Moreover, CAT activity notably decreased in *GhN/AINV23*-silenced plants (Fig. 5C), whereas MDA content significantly increased (Fig. 5D). These findings imply that inhibiting *GhN/AINV23* expression reduces drought tolerance in cotton.

## Discussion

Neutral/alkaline invertase plays a crucial role in plant growth, development, and stress resistance [11–13, 15–18]. This research evaluates the characteristics of *GhN/AINV23* and its function in plant growth and drought resistance. The results demonstrate that GhN/AINV23 is localized in the cytoplasm and cell membrane and can improve plant growth and drought resistance.

### GhN/AINV23 promotes vegetative phase change by hydrolyzing sucrose

Vegetative phase transition refers to the transition from the juvenile to adult vegetative phase. In *Arabidopsis*, increase of leaf length/width ratio is one of the markers of vegetative phase change [45]. Meng (2021) demonstrated that the activity of CINV1 is controlled by a glucose feed-forward loop that converts sucrose into glucose signals to dynamically control the transition from juvenile to adult [46]. In our research, the phylogenetic tree showed that GhN/AINV23 was closely related to CINV1. *GhN/AINV23* overexpression increased the leaf length/width ratio, invertase activity and glucose content in *Arabidopsis*. This suggests that *GhN/AINV23* may have a similar function to *CINV1*, and may promote vegetative growth by hydrolyzing sucrose to increase glucose content.

### GhN/AINV23 participates in the regulation of sucrose metabolism and root development

Appropriate amounts of glucose can promote root development [7], whereas high concentrations of exogenous glucose can inhibit root meristem growth [8]. The neutral-alkaline invertase can irreversibly convert sucrose into glucose and fructose [10]. This indicates that the neutral-alkaline invertase can generate glucose signals by converting sucrose and regulate root development. Lack of function of cinv1 and cinv2 leads to abnormal sucrose and glucose levels and inhibited primary root growth [14]. CINV1 was also found to regulate plant primary root growth by glucose signaling [46]. Overexpression of *GhN/AINV23* in *Arabidopsis* promoted root development and increased endogenous glucose content. Treatment with sucrose or 1% glucose significantly enhanced root development in transgenic Arabidopsis. However, with the increase of exogenous glucose content, root development of transgenic *Arabidopsis* was inhibited. This indicates that when glucose content exceeds a certain threshold, there will be negative feedback to overexpression *GhN/AINV23 Arabidopsis* root development, suggesting that *GhN/AINV23* is involved in root development in the regulation of sucrose metabolism, and this regulation is also regulated by glucose content feedback.

### GhN/AINV23 positively regulates plant drought resistance

Drought stress remains a significant limiting factor for crop yields [47, 48]. It affects cell water potential, dilatation, and photosynthesis, leading to wilting and potentially plant death [49–51]. Carbon fixation can decrease under stress conditions, such as drought, but plants accumulate large amounts of soluble sugars, such as sucrose [52], which act as osmoregulatory substances to maintain basic cellular structure and function by retaining water [53]. Regulation of sucrose metabolism is typically mediated by neutral/alkaline invertases [10]. Previous studies have demonstrated that N/AINVs can positively regulate plant drought resistance by regulating osmotic pressure and reactive oxygen content [13, 23]. In addition, among the known plant hormones, ABA is the most important hormone involved in mediating plant drought response [54]. Our results demonstrate that the expression of *GhN/AINV23* is significantly induced by ABA and involved in sucrose metabolism. Transgenic *Arabidopsis* and VIGS experiments suggest that GhN/AINV23 positively regulates drought resistance. This suggests that GhN/AINV23 regulates drought resistance by participating in sucrose metabolism, although further investigation is required. In short, our study expands the understanding of neutral/alkaline invertases and provides a candidate gene for improving drought resistance in cotton.

## Conclusion

This study provides in-depth analysis of the characteristics and function of *GhN/AINV23*, including its sequence characterization, subcellular localization, expression analysis, and biological function. Our findings demonstrate that GhN/AINV23 is localized in the cytoplasm and cell membrane and plays a role in sucrose metabolism. Further studies found that GhN/AINV23 promote vegetative phase change and increase drought resistance in plants, and promote root development by regulating sucrose metabolism. These results lay the groundwork for future improvements in cotton fiber yield by utilizing GhN/AINV23.

### Electronic supplementary material

Below is the link to the electronic supplementary material.

Additional file 1: Table S1: Oligonucleotide primers used in this study

Additional file 2: Figure S1: Bioinformatics analysis of GhN/AINV23. Three-dimensional GhN/AINV23 model (**A**). Phylogenetic tree of GhN/AINV23 and Arabidopsis neutral/alkaline invertases (**B**). Amino acid sequence consistency analysis of GhN/AINV23, AtCINV1 and AtCINV2 (**C**). The cis-element analysis of GhN/AINV23 promoter region (**D**)

Additional file 2: Figure S2: Observation of drought treatment on WT plants and transgenic lines at maturity stage. Phenotype before drought treatment (**A**), phenotype after 15 days of drought treatment (**B**), phenotype after rehydration for 7 days (**C**)

## Data Availability

Please contact author for data requests.

## Abbreviations

3D model: Three Dimensional model
ABA: Abscisic acid
AS: Acetosyringone
AINV: Acid invertase
CAT: Catalase
CDS: Coding sequence
MDA: Malondialdehyde
MW: Molecular weight
N/AINV: Neutral/alkaline invertase
PIP5K9: Phosphatidylinositol monophosphate 5-kinase 9
qRT-PCR: Quantitative real-time polymerase chain reaction
TRV: Tobacco rattle virus
VIGS: Virus-induced gene silencing

## References

[1] Lastdrager J, Hanson J, Smeekens S (2014). Sugar signals and the control of plant growth and development. J Exp Bot.

[2] Jeandet P, Formela-Luboińska M, Labudda M, Morkunas I (2022). The role of sugars in plant responses to stress and their regulatory function during development. Int J Mol Sci.

[3] Ruan YL, Jin Y, Yang YJ, Li GJ, Boyer JS (2010). Sugar input, metabolism, and signaling mediated by invertase: roles in development, yield potential, and response to drought and heat. Mol Plant.

[4] Wind J, Smeekens S, Hanson J (2010). Sucrose: metabolite and signaling molecule. Phytochemistry.

[5] Sakr S, Wang M, Dédaldéchamp F, Perez-Garcia MD, Ogé L, Hamama L, Atanassova R (2018). The sugar-signaling hub: overview of regulators and interaction with the hormonal and metabolic network. Int J Mol Sci.

[6] Freixes S, Thibaud MC, Tardieu F, Muller B (2002). Root elongation and branching is related to local hexose concentration in *Arabidopsis thaliana* seedlings. Plant Cell Environ.

[7] Mishra BS, Singh M, Aggrawal P, Laxmi A (2009). Glucose and auxin signaling interaction in controlling *Arabidopsis thaliana* seedlings root growth and development. PLoS ONE.

[8] Yuan TT, Xu HH, Zhang KX, Guo TT, Lu YT (2014). Glucose inhibits root meristem growth via ABA INSENSITIVE 5, which represses PIN1 accumulation and auxin activity in *Arabidopsis*. Plant Cell Environ.

[9] Giehl RF, Gruber BD, von Wirén N (2014). It’s time to make changes: modulation of root system architecture by nutrient signals. J Exp Bot.

[10] Sturm A, Invertases (1999). Primary structures, functions, and roles in plant development and sucrose partitioning. Plant Physiol.

[11] Vargas WA, Salerno GL (2010). The Cinderella story of sucrose hydrolysis: Alkaline/neutral invertases, from cyanobacteria to unforeseen roles in plant cytosol and organelles. Plant Sci.

[12] Li W, Liu Y, Liu M, Zheng Q, Li B, Li Z, Li H (2019). Sugar accumulation is associated with leaf senescence induced by long-term high light in wheat. Plant Sci.

[13] Qi X, Wu Z, Li J, Mo X, Wu S, Chu J, Wu P (2007). AtCYT-INV1, a neutral invertase, is involved in osmotic stress-induced inhibition on lateral root growth in *Arabidopsis*. Plant Mol Biol.

[14] Barratt DHP, Derbyshire P, Findlay K, Pike M, Wellner N, Lunn J, Feil R, Simpson C, Maule AJ, Smith AM (2009). Normal growth of *Arabidopsis* requires cytosolic invertase but not sucrose synthase. Proc Natl Acad Sci U S A.

[15] Samac DA, Bucciarelli B, Miller SS, Yang SS, O’Rourke JA, Shin S, Vance CP (2015). Transgene silencing of sucrose synthase in alfalfa (*Medicago sativa* L.) stem vascular tissue suggests a role for invertase in cell wall cellulose synthesis. BMC Plant Biol.

[16] Vargas WA, Pontis HG, Salerno GL (2007). Differential expression of alkaline and neutral invertases in response to environmental stresses: characterization of an alkaline isoform as a stress-response enzyme in wheat leaves. Planta.

[17] Welham T, Pike J, Horst I, Flemetakis E, Katinakis P, Kaneko T, Sato S, Tabata S, Perry J, Parniske M (2009). A cytosolic invertase is required for normal growth and cell development in the model legume, *Lotus japonicus*. J Exp Bot.

[18] Lou Y, Gou JY, Xue HW (2007). PIP5K9, an *Arabidopsis* phosphatidylinositol monophosphate kinase, interacts with a cytosolic invertase to negatively regulate sugar-mediated root growth. Plant Cell.

[19] Wang YJ, Zhen XH, Zhou YJ, Wang YL, Hou JY, Wang X, Li RM, Liu J, Hu XW, Geng MT, Yao Y, Guo JC (2022). *MeNINV1*: an alkaline/neutral invertase gene of *Manihot esculenta*, enhanced sucrose catabolism and promoted plant vegetative growth in transgenic *Arabidopsis*. Plants.

[20] Cheng LT, Jin JJ, He XX, Luo ZP, Wang Z, Yang J, Xu X (2023). Genome-wide identification and analysis of the invertase gene family in tobacco (*Nicotiana tabacum*) reveals NtNINV10 participating the sugar metabolism. Front Plant Sci.

[21] Huang W, Li Y, Du Y, Pan L, Huang Y, Liu H, Zhao Y, Shi Y, Ruan YL, Dong Z, Jin W (2022). Maize cytosolic invertase INVAN6 ensures faithful meiotic progression under heat stress. New Phytol.

[22] Wang C, Wang GH, Wen XY, Qu XJ, Zhang YY, Zhang XY, Deng PC, Chen CH, Ji WQ, Zhang H (2023). Characteristics and expression analysis of Invertase Gene Family in Common Wheat (*Triticum aestivum* L). Genes.

[23] Xu W, Cui K, Xu A, Nie L, Huang J, Peng S (2015). Drought stress condition increases root to shoot ratio via alteration of carbohydrate partitioning and enzymatic activity in rice seedlings. Acta Physiol Plant.

[24] Dahro B, Wang Y, Khan M, Zhang Y, Fang T, Ming R, Li C, Liu JH (2022). Two AT-Hook proteins regulate *A/NINV7* expression to modulate sucrose catabolism for cold tolerance in *Poncirus trifoliata*. New Phytol.

[25] Xiang L, Li Y, Rolland F, Van den Ende W (2011). Neutral invertase, hexokinase and mitochondrial ROS homeostasis: emerging links between sugar metabolism, sugar signaling and ascorbate synthesis. Plant Signal Behav.

[26] Xiang L, Le Roy K, Bolouri-Moghaddam M-R, Vanhaecke M, Lammens W, Rolland F, Van den Ende W (2011). Exploring the neutral invertase–oxidative stress defence connection in *Arabidopsis thaliana*. J Exp Bot.

[27] Dahro B, Wang F, Peng T, Liu JH (2016). *PtrA/NINV*, an alkaline/neutral invertase gene of *Poncirus trifoliata*, confers enhanced tolerance to multiple abiotic stresses by modulating ROS levels and maintaining photosynthetic efficiency. BMC Plant Biol.

[28] Liu J, Han L, Huai B, Zheng P, Chang Q, Guan T, Li D, Huang L, Kang Z (2015). Down-regulation of a wheat alkaline/neutral invertase correlates with reduced host susceptibility to wheat stripe rust caused by *Puccinia Striiformis*. J Exp Bot.

[29] Jia L, Zhang B, Mao C, Li J, Wu Y, Wu P, Wu Z (2008). OsCYT-INV1 for alkaline/neutral invertase is involved in root cell development and reproductivity in rice (*Oryza sativa* L). Planta.

[30] Pignocchi C, Ivakov A, Feil R, Trick M, Pike M, Wang TL, Lunn JE, Smith AM (2021). Restriction of cytosolic sucrose hydrolysis profoundly alters development, metabolism, and gene expression in *Arabidopsis* roots. J Exp Bot.

[31] Zhang HB, Li Y, Wang B, Chee PW. Recent advances in cotton genomics. Int J Plant Genomics. 2008; 2008:1–20. 10.1155/2008/742304 PMC2233810 18288253

[32] Liu B, Wang X, Li K, Cai Z (2021). Spatially resolved metabolomics and lipidomics reveal salinity and drought-tolerant mechanisms of cottonseeds. J Agric Food Chem.

[33] Chen BZ, Wang XY, Lv JY, Ge MJ, Qiao KK, Chen QJ, Zhang KP, Wang JS, Fan SL, Ma QF (2021). GhN/AINV13 positively regulates cotton stress tolerance by interacting with the 14-3-3 protein. Genomics.

[34] Zhu T, Liang C, Meng Z, Sun G, Meng Z, Guo S, Zhang R (2017). CottonFGD: an integrated functional genomics database for cotton. BMC Plant Biol.

[35] Kumar S, Stecher G, Tamura K (2016). MEGA7. Molecular evolutionary genetics analysis version 7.0 for bigger datasets. Mol Biol Evol.

[36] Lescot M, Déhais P, Thijs G, Marchal K, Moreau Y, Van de Peer Y, Rouzé P, Rombauts S (2002). PlantCARE, a database of plant cis-acting regulatory elements and a portal to tools for in silico analysis of promoter sequences. Nucleic Acids Res.

[37] Chen C, Chen H, Zhang Y, Thomas HR, Frank MH, He Y, Xia R (2020). TBtools: an integrative toolkit developed for interactive analyses of big biological data. Mol Plant.

[38] Huang YW, Nie YX, Wan YY, Chen SY, Sun Y, Wang XJ, Bai J (2013). Exogenous glucose regulates activities of antioxidant enzyme, soluble acid invertase and neutral invertase and alleviates dehydration stress of cucumber seedlings. Sci Hortic-amsterdam.

[39] Fils-Lycaon B, Julianus P, Chillet M, Galas C, Galas O, Rinaldo D, Mbéguié-A-Mbéguié D (2011). Acid invertase as a serious candidate to control the balance sucrose versus (glucose + fructose) of banana fruit during ripening. Sci Hortic-amsterdam.

[40] Kabasakalian P, Kalliney S, Westcott A (1974). Enzymatic blood glucose determination by colorimetry of N, N-diethylaniline-4-aminoantipyrine. Clin Chem.

[41] Varandas S, Teixeira MJ, Marques JC, Aguiar A, Aguiar A, Bastos M (2004). Glucose and fructose levels on grape skin: interference in *Lobesia botrana* behaviour. Anal Chim Acta.

[42] Livak KJ, Schmittgen TD (2001). Analysis of relative gene expression data using real-time quantitative PCR and the 2^–∆∆CT^ method. Methods.

[43] Burch-Smith TM, Anderson JC, Martin GB, Dinesh-Kumar SP (2004). Applications and advantages of virus-induced gene silencing for gene function studies in plants. Plant J.

[44] Yu CS, Chen YC, Lu CH, Hwang JK (2006). Prediction of protein subcellular localization. Proteins.

[45] Usami T, Horiguchi G, Yano S, Tsukaya H (2009). The *more and smaller cells* mutants of *Arabidopsis* thaliana identify novel roles for *SQUAMOSA PROMOTER BINDING PROTEIN-LIKE* genes in the control of heteroblasty. Development.

[46] Meng LS, Bao QX, Mu XR, Tong C, Cao XY, Huang JJ, Xue LN, Liu CY, Fei Y, Loake GJ (2021). Glucose- and sucrose-signaling modules regulate the *Arabidopsis* juvenile-to-adult phase transition. Cell Rep.

[47] Abrams MD (1990). Adaptations and responses to drought in *Quercus* species of North America. Tree Physiol.

[48] Allen CD, Macalady AK, Chenchouni H, Bachelet D, McDowell N, Vennetier M, Kitzberger T, Rigling A, Breshears DD, Hogg EH (2010). A global overview of drought and heat-induced tree mortality reveals emerging climate change risks for forests. For Ecol Manag.

[49] Gupta A, Rico-Medina A, Caño-Delgado AI (2020). The physiology of plant responses to drought. Science.

[50] Ahluwalia O, Singh PC, Bhatia R (2021). A review on drought stress in plants: implications, mitigation and the role of plant growth promoting rhizobacteria. Resour Environ Sustain.

[51] Reddy AR, Chaitanya KV, Vivekanandan M (2004). Drought-induced responses of photosynthesis and antioxidant metabolism in higher plants. J Plant Physiol.

[52] Tauzin AS, Giardina T (2014). Sucrose and invertases, a part of the plant defense response to the biotic stresses. Front Plant Sci.

[53] Cushman JC (2001). Osmoregulation in plants: implications for agriculture1. Amer Zool.

[54] Davies WJ, Kudoyarova G, Hartung W (2005). Long-distance ABA signaling and its relation to other signaling pathways in the detection of soil drying and the mediation of the plant’s response to drought. J Plant Growth Regul.

