# Supplementary figures and images for: Exploring the role of GhN/AINV23: implications for plant growth, development, and drought tolerance
Source: Biol Direct. 2024 Mar 14;19:22. doi: 10.1186/s13062-024-00465-2 (PMC10938729; doi:10.1186/s13062-024-00465-2)

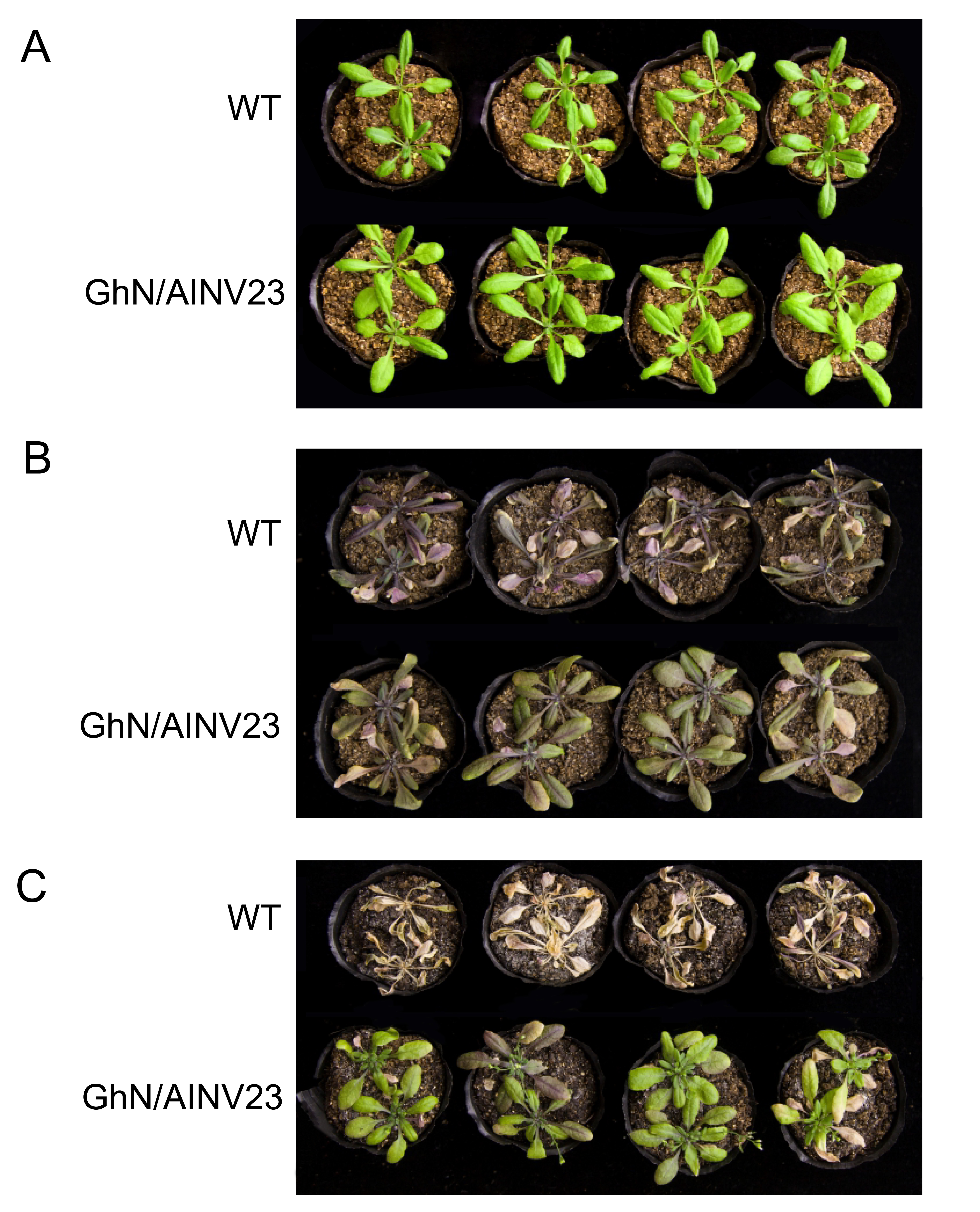

Supplement: Supplementary file 3 — Additional file 2: Figure S2: Observation of drought treatment on WT plants and transgenic lines at maturity stage. Phenotype before drought treatment (A), phenotype after 15 days of drought treatment (B), phenotype after rehydration for 7 days (C) [file 13062_2024_465_MOESM3_ESM.jpg]
